# Using Decision Trees to Examine Environmental and Behavioural Factors Associated with Youth Anxiety, Depression, and Flourishing

**DOI:** 10.3390/ijerph191710873

**Published:** 2022-08-31

**Authors:** Katelyn Battista, Karen A. Patte, Liqun Diao, Joel A. Dubin, Scott T. Leatherdale

**Affiliations:** 1 School of Public Health Sciences, University of Waterloo, Waterloo, ON N2L 3G1, Canada; 2 Department of Health Sciences, Brock University, St. Catharines, ON L2S 3A1, Canada; 3 Department of Statistics and Actuarial Science, University of Waterloo, Waterloo, ON N2L 3G1, Canada

**Keywords:** decision trees, mental health, youth, school climate, home environment

## Abstract

Modifiable environmental and behavioural factors influence youth mental health; however, past studies have primarily used regression models that quantify population average effects. Decision trees are an analytic technique that examine complex relationships between factors and identify high-risk subgroups to whom intervention measures can be targeted. This study used decision trees to examine associations of various risk factors with youth anxiety, depression, and flourishing. Data were collected from 74,501 students across Canadian high schools participating in the 2018–2019 COMPASS Study. Students completed a questionnaire including validated mental health scales and 23 covariates. Decision trees were grown to identify key factors and subgroups for anxiety, depression, and flourishing outcomes. Females lacking both happy home life and sense of connection to school were at greatest risk for higher anxiety and depression levels. In contrast with previous literature, behavioural factors such as diet, movement and substance use did not emerge as differentiators. This study highlights the influence of home and school environments on youth mental health using a novel decision tree analysis. While having a happy home life is most important in protecting against youth anxiety and depression, a sense of connection to school may mitigate the negative influence of a poor home environment.

## 1. Introduction

Mental illness has garnered increased global concern in recent years as a leading contributor to global disease burden [1,2]. Youth have been identified as a priority group for addressing mental health concerns [3,4], given that the onset of mental illness primarily occurs during adolescence [5] and untreated mental illness during adolescence can lead to negative consequences in adulthood [6]. Depression and anxiety are among the mental illnesses associated with highest suicide risk [7], and have also been associated with increased substance use during adolescence [8,9]. While previous efforts around youth mental health have primarily focused on combating mental illnesses such as anxiety and depression, recent approaches have also emphasized the importance of enhancing mental well-being [10,11,12]. Flourishing, defined as a state of psychosocial well-being, has been associated with increased life expectancy [13]. Among youth, flourishing has also been associated with lower likelihood of substance use [8,9,14] and improved academic performance [15,16,17].

Following Bronfenbrenner’s social-ecological model [18], the causal mechanisms driving mental illness onset in youth involve complex interactions between a hierarchical network of individual (e.g., genetic, biological) and environmental (e.g., interpersonal, organizational, community, public policy) factors. Past studies have found widely varying estimates of the proportion of mental illness onset attributable to genetic vs. environmental influences: anywhere from 15% to 80% of youth-onset depression [19] and 18% to 35% of youth-onset anxiety [20] is heritable, with the remaining attributable to environmental factors. Genetic and environmental influences on flourishing are less understood, though one past study examining related well-being constructs found heritability estimates of 34% for subjective happiness and 44% for life satisfaction [21]. Thus, while there is evidence of a genetic component to youth mental illness and well-being, the contextual environment plays an influential role.

From a public health perspective, the contextual environment is important as many environmental risk and protective factors can be considered modifiable and hence potential intervention leverage points. The importance of context on youth mental health outcomes is recognized within national public policy guidance. The Mental Health Strategy for Canada [22] published by the Mental Health Commission of Canada (MHCC) prioritizes support for youth mental health with calls to “increase the capacity of families, caregivers, schools, post-secondary institutions and community organizations”. Publicly funded community- and school-based supports can act as universal access points for prevention and early intervention efforts and are consistently highlighted as pillars in federal [12,22] and provincial mental health strategies. Related interpersonal factors such as family relationships [23,24,25], peer relationships [26,27], bullying [28], and school connectedness [23,27,29] have previously been linked to youth mental health outcomes. Previous research has also found associations to modifiable behavioural factors such as diet [30], movement behaviours [30,31,32], sleep [33], and substance use [34]. However, two major limitations of past studies are that associations to domains of risk and protective factors are generally examined in isolation, and that the primarily regression-based analytic methods focus on quantifying average effects across the study population without consideration for potential high-risk subgroups.

Decision trees are a machine learning-based analytic technique comprising several classes of modeling algorithms [35], which group similar subjects with respect to an outcome using a tree structure. While more commonly used in medical screening and diagnostics for disease prediction, decision trees have seen recent increasing use in public health research [36] to examine complex relationships between outcomes and risk factors and identify high-risk subgroups to whom prevention and intervention measures can be targeted. Decision trees have previously been used to examine depression outcomes in various adult populations; past studies involving various environmental factors have found social connection [37] and aspects of financial stability [37,38] to be important, while substance use was only found to be a risk factor among certain subgroups [38]. However, youth face distinct contextual risk factors, and previous research using decision trees to examine youth mental health is limited. Seely et al. [39] examined major depressive disorder (MDD) onset among adolescent females and found that the subgroup of previously depressed females with poor school functioning was at greatest risk for MDD onset, while family support was only a protective factor among the subgroup of females without previous depressive symptoms. Hill et al. [40] found friend support to be a protective factor against the development of MDD among those with subclinical symptoms, while subgroups with history of anxiety and substance use disorder were at higher risk. These studies highlight the importance of interpersonal factors (school and family support) and behavioural factors (substance use); however, sample sizes in both studies were small. To our knowledge, no previous studies have used decision trees to examine anxiety or flourishing outcomes among youth.

Given the importance of environmental factors on youth mental health, the purpose of this study is therefore to use decision tree analysis to examine associations of modifiable behavioural and interpersonal risk factors with youth anxiety, depression, and flourishing, with a focus on characterizing groups at highest risk of mental ill-health. Results of this exploratory analysis are contrasted against those of traditional regression-based analysis and compared to findings from previous literature to highlight the unique insights gleaned from decision tree analysis.

## 2. Materials and Methods

### 2.1. Study Design and Sample

COMPASS is a prospective cohort study (2012–2021) designed to examine the impact of policies and environmental characteristics on Canadian secondary school students [41]. COMPASS collects data on multiple health behaviours and risk factors including mental health, substance use, healthy eating, movement behaviours, bullying and academics. Additional details about the COMPASS study design and methods are available in print [41] and online (https://uwaterloo.ca/compass-system accessed on 13 July 2022). The COMPASS study received ethics clearance from the University of Waterloo Research Ethics Board (ORE 30118) and participating school boards.

The current study uses student-level data from 2018–2019 (Year 7) of the COMPASS Study. The sample consists of 74,501 students from 136 schools in Ontario (61 schools), Alberta (8 schools), British Columbia (15 schools) and Quebec (52 schools). Schools were purposefully recruited into the COMPASS study according to their use of active-information, passive-consent protocols, which have been shown to be important for collecting unbiased data among youth [42]. Further details on general school recruitment procedures [43] and 2018–2019 sample recruitment [44] are available. All students within a recruited school who received passive parental permission [41] were invited to participate, and students could withdraw at any time. The participation rate for 2018–2019 was 81.9%, with the primary reason for non-participation being absenteeism at the time of data collection.

### 2.2. Measures

#### 2.2.1. Compass Student Questionnaire

The COMPASS student questionnaire is an anonymized, self-administered, paper-based questionnaire. The questionnaire is completed during class time and takes approximately 40 min to complete. Data collection procedures for student questionnaire administration are documented [45]. This study examined three mental health scale outcomes measuring anxiety, depression, and flourishing, as well as 23 predictor measures related to questionnaire items on demographics, body weight, healthy eating, movement behaviours, substance use, bullying, academics, and perceived school, family, and friend support.

#### 2.2.2. Mental Health Outcome Measures

Depression is measured using the Centre of Epidemiologic Studies Depression Scale 10—Revised (CESD-10) [46,47]. The CESD-10 is measured as a continuous score ranging from 0 to 30, with higher scores indicating greater degrees of depressive symptomatology, and scores at or above 10 indicating clinically relevant depressive symptoms [46]. Anxiety is measured using the Generalized Anxiety Disorder 7-item Scale (GAD-7) [48]. The GAD-7 is measured as a numeric score ranging from 0 to 21, with higher scores indicating greater levels of anxiety, and scores at or above 10 indicating clinically relevant anxiety symptoms [48]. Flourishing is measured using a modified version of Diener’s Flourishing Scale (FS) [49]. The FS is a numeric score ranging from 8 to 40 with higher scores indicating greater levels of flourishing. Consistent with recommendations for Likert-style scales [50,51] all individual mental health scale items were person-mean imputed for students missing 1 or 2 items. Students missing three or more scale items on the GAD-7, CESD-10, or FS outcomes were not found to be significantly different on any predictor measures from students missing two or fewer values and were therefore excluded from the respective analyses.

#### 2.2.3. Predictor Measures

##### Demographics

Students are asked to indicate their sex (male, female) and age (12 to 18 years). Students self-identify their ethnicity with options for White, Black, Asian, Hispanic, and Other, with the option to select multiple ethnicities. Weekly spending money is measured as a proxy for socioeconomic status, with options ranging from “$0” to “More than $100”.

##### Weight Status and Perception

Students are asked how they describe their weight with options for Slightly/Very Underweight, About the Right Weight, or Slightly/Very Overweight. An objective measure of Body Mass Index (BMI) is calculated based on self-reported height and weight, and classified into Underweight, Normal Weight, Overweight, or Obese based on World Health Organization age- and sex-adjusted cut-offs. Students with missing height or weight data are included in a separate Not Stated category due to the tendency for BMI data to have non-random missingness mechanisms [52].

##### Diet and Eating Behaviours

Students are asked whether they eat breakfast daily and their number of daily servings of fruits and vegetables.

##### Movement Behaviours

Daily moderate-to-vigorous physical activity is measured by asking students the amount and the intensity of activity performed on each of the last seven days. Total daily screen time is measured by asking students the amount of time they usually spend texting/messaging/emailing, playing video/computer games, talking on the phone, watching TV/movies and surfing the internet. Daily sleep time is also measured by asking how much time they usually spend sleeping. These measures have been shown to have moderate validity when compared to objective measures and high test–retest reliability [53].

##### Substance Use

Current use of cigarettes and e-cigarettes is measured based on students indicating any use in the last 30 days. Current use of cannabis is measured based on use at least once a month in the past 12 months. Current binge drinking is measured based on having five or more drinks at least once a month in the past 12 months.

##### Bullying and Academics

Bullying is measured using two indicators of whether students have been bullied or have bullied others in the past 30 days. Academic expectations are measured based on students indicating expectation to attend some form of post-secondary education. Truancy is measured based on the number of classes skipped in the past four weeks.

##### School Connectedness

School connectedness (SC) is measured using an adapted version of the National Longitudinal Study of Adolescent Health SCS-5 item scale [54]. The SC scale is as a numeric score ranging 6 to 24, with higher scores indicating greater SC. Scale items include the SCS-5 measures “I feel close to people at my school”, “I feel I am part of my school”, “I am happy to be at my school”, “I feel the teachers at my school treat me fairly”, and “I feel safe in my school”, and an additional measure “Getting good grades is important to me”, with response options ranging from Strongly Agree to Strongly Disagree.

##### Social Support

Family and friend support are measured based on three individual items from the Multidimensional Scale of Perceived Social Support [55]. Students are asked to indicate level of agreement with the statements “I have a happy home life”, “I can talk about my problems with my family”, and “I can talk about my problems with my friends”.

#### 2.2.4. School-Level Census Data

Province and school enrolment size are recorded for each participating school. School area median income and school urbanicity are measured by linking to Statistics Canada 2016 Census data based on each school’s forward sortation area [56,57].

### 2.3. Analysis

Mixed effects regression trees were separately grown for GAD-7, CESD-10 and FS outcomes including all predictor variables. Random Effects EM (RE-EM) trees were used following the algorithm proposed by Sela and Simonoff [58] and Hajjem [59] to account for school-level clustering based on the assumption that students from the same school may have greater similarity in responses than students from different schools. Students with missing values on a given outcome were therefore excluded from the analysis, while missing predictor values were included and accounted for using surrogate splitting. Given the large sample size, a splitting rule was set requiring a minimum increase to adjusted R-squared (R^2^_adj_) of 0.005 to limit splits that would be unlikely to improve overall prediction accuracy. Tree pruning using 10-fold cross-validation was performed to limit overfitting to the sample data. The smallest tree within one standard deviation of the minimum cross-validation error was chosen. The R software was used for all analyses [60]; package “REEMtree” [61] was used to grow the trees, and the package “rpart.plot” [62] was used for plotting.

To provide a comparison of the RE-EM tree results, linear mixed effects regression (LME) models were also fit for each outcome including all predictor variables, using the R package “nlme” [63]. Students with missing values on a given outcome or any predictors were excluded from the analysis; maximum likelihood estimation is used within LME to account for missing at random data. A random intercept term was included to account for school-level clustering. Backward variable selection was implemented based on Akaike’s Information Criterion (AIC). Intraclass correlation coefficients (ICCs) were calculated on null LME models to quantify the amount of variability in mental health outcomes that can be attributed to differences between schools.

## 3. Results

### 3.1. Sample Characteristics

Sample characteristics are shown in Table 1. The mean GAD-7 score in the sample was 6.2 (SD 5.6) with 24.0% of the sample having scores of 10 or higher, which indicates clinically relevant anxiety symptoms. The mean CESD-10 score was 8.8 (SD 6.1) with 37.0% of the sample having scores of 10 or higher, indicating clinically relevant depressive symptoms. The average FS score was 32.2 (SD 5.7). The sample was 49.1% female with mean age 15.2 (SD 1.5) and predominantly identified as white (68.5%). ICCs showed modest between-school variability of 3.35% in student GAD-7 scores, 2.12% in CESD-10 scores, and 4.29% in FS scores.

### 3.2. GAD-7

**Figure 1:**
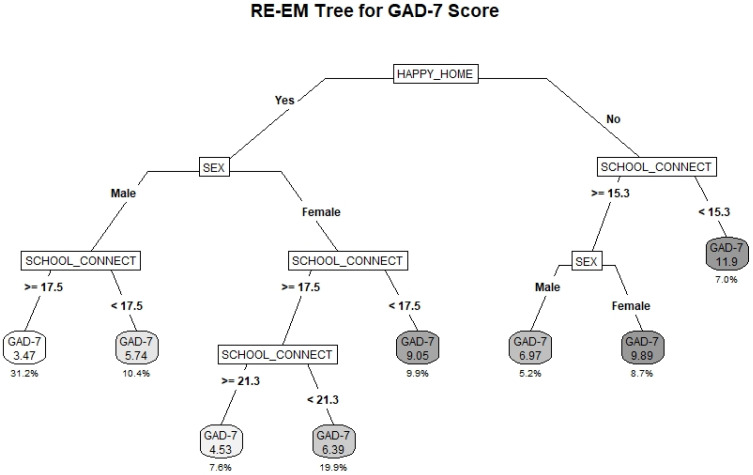
RE-EM Tree predicting average GAD-7 score for students participating in Year 7 (2018–2019) of the COMPASS Study (*N* = 71.736). The GAD-7 score represents the average scale score within the subgroup. The percentage below represents the total percentage of the sample comprised by the subgroup.

The RE-EM tree fitted to the GAD-7 outcome is provided in Figure 1. The R^2^_adj_ for the model was 0.23. Having a happy home life was identified as the primary splitting factor; that is, the factor that best distinguishes between high and low GAD-7 scores. Among students without a happy home life, school connectedness (SC) was identified as a protective factor. The highest risk subgroup comprised students without a happy home life and with low SC (score < 15.3); the average GAD-7 score in this group was 11.9, which is above the threshold of 10 for having clinically relevant anxiety symptoms. This subgroup constituted 7% of the total sample. Among students with higher SC (score ≥ 15.3) females had average GAD-7 scores nearly 3 points higher than their male counterparts (9.89 compared to 6.97), closely approaching the clinical threshold.

Among those with a happy home life, sex was identified as a key differentiating factor; however, SC was a protective factor for both males and females. Both subgroups of males with high and low SC had lower average GAD-7 scores than females, except for the small subgroup of females with very high SC scores. Notably, females with low SC (score < 17.5) had much higher average GAD-7 scores than their male counterparts (9.07 compared to 5.74). The largest final subgroup comprised males with high SC who indicated having a happy home life (31.2% of sample), and this group had the lowest average GAD-7 score of 3.47.

The LME model for GAD-7 score is provided in Table 2. The R^2^_adj_ for the model was 0.32. Consistent with the RE-EM tree, having a happy home life (Est. −1.96 [−2.07,−1.85]), male sex (Est. −2.57 [−2.65,−2.49]), and higher SC (Est. −0.32 [−0.33,−0.30] per unit) were significantly associated with lower GAD-7 scores. Additionally, 18 other covariates were found to have some magnitude of significant association, likely due to the large sample size. Notably, being bullied in the past 30 days was associated with higher GAD-7 scores (Est. 1.92 [1.79,2.05]).

### 3.3. CESD-10

**Figure 2:**
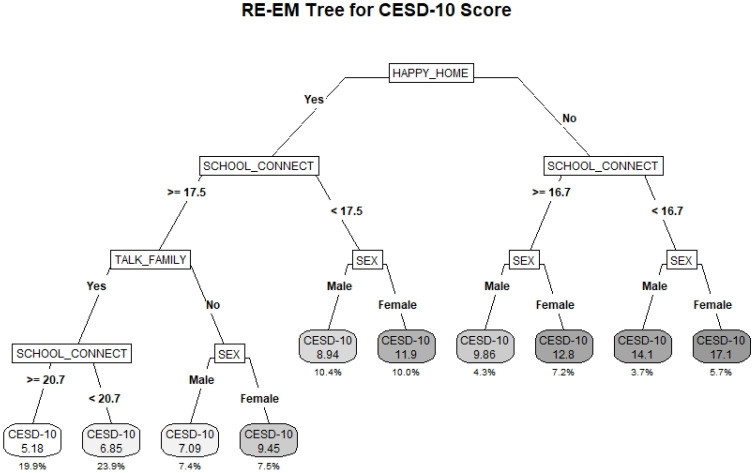
RE-EM Tree predicting average CESD-10 score for students participating in Year 7 (2018–2019) of the COMPASS Study (*N* = 70,610). The CESD-10 score represents the average scale score within the subgroup. The percentage below represents the total percentage of the sample comprised by the subgroup.

The RE-EM tree fitted to the CESD-10 outcome is provided in Figure 2. The R^2^_adj_ for the model was 0.30. Like the GAD-7 tree, having a happy home life was identified as the primary splitting factor. Among those without a happy home life, SC was the most important factor, followed by sex, with the highest risk subgroup comprising females without a happy home life and with low SC (average CESD-10 score 16). Among both subgroups with low and high SC, males had lower average CESD-10 scores than females. Notably, the average CESD-10 score met or exceeded the threshold for clinically relevant depressive symptoms of 10 or higher among all subgroups without a happy home life.

Among those with a happy home life, SC was again the most important factor. Females with a happy home life but low SC (score < 17.5) had an average CESD-10 score of 11.9, exceeding the threshold for clinically relevant depressive symptoms. Students of both sexes with a happy home life were further differentiated by whether they felt comfortable talking about problems with their family. Among those who did not feel comfortable, females had higher average CESD-10 scores than males. Those who did feel comfortable were further split based on having very high very high SC, with those students having the lowest average CESD-10 score of 5.18, followed by those with moderately high SC scores who had an average CESD-10 score of 6.85. Notably, being able to talk about problems with family was identified as a protective factor only among the subgroup of students with a happy home life and high SC.

The LME model for CESD-10 score is provided in Table 2. The R^2^_adj_ for the model was 0.39. Consistent with the RE-EM tree, having a happy home life (Est. −2.75 [−2.86,−2.64]), higher SC (Est. −0.45 [−0.46,−0.43] per unit), male sex (Est. −2.11 [−2.19,−2.03]), and feeling able to talk about problems with family (Est. −1.31 [−1.40,−1.22]) were significantly associated with lower CESD-10 scores. Additionally, 17 other covariates were found to have some magnitude of significant association. Like the GAD-7 outcome, being bullied in the past 30 days was associated with higher CESD-10 scores (Est. 2.05 [1.93,2.18]).

### 3.4. Flourishing Scale

**Figure 3:**
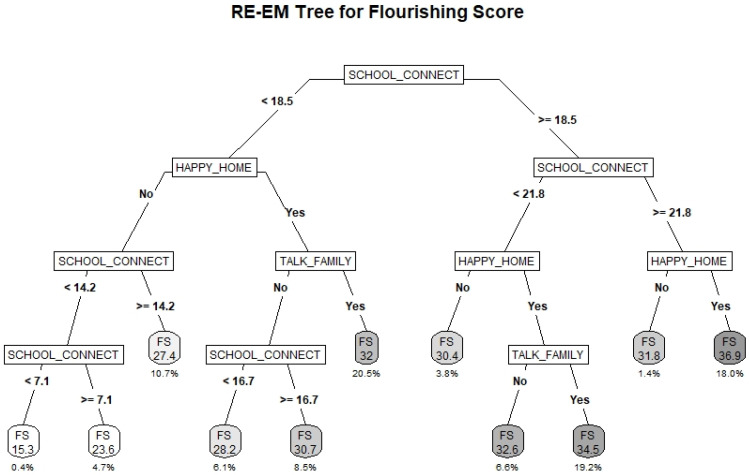
RE-EM Tree predicting average FS score for students participating in Year 7 (2018–2019) of the COMPASS Study (*N* = 72,415). The FS score represents the average scale score within the subgroup. The percentage below represents the total percentage of the sample comprised by the subgroup.

The RE-EM tree fitted to the FS outcome is provided in Figure 3. The R^2^_adj_ for the model was 0.42. The primary splitting variable is SC score. Among those with both moderately high and very high SC, having a happy home life was identified as the next most important factor. Students with very high SC and a happy home life had the highest average flourishing score of 36.9. Among students with moderately high SC and a happy home life, those who felt able to talk about problems with family had higher FS scores than those without (34.55 vs. 32.6), though this factor was not identified as important among students who did not already have a happy home life.

Among those with low SC, having a happy home life was again identified as the most important factor, and being able to talk about problems with family was identified as important among those with a happy home life. The tree further differentiated subgroups by SC among those either without a happy home life or who felt unable to talk about problems with family. The highest risk subgroups comprised students without a happy home life and with low or very low SC, having average FS scores of 23.8 and 15.3, respectively.

The LME model for FS score is provided in Table 2. The R^2^_adj_ for the model was 0.51. Consistent with the RE-EM tree, higher SC (Est. 0.68 [0.67,0.69] per unit), having a happy home life (Est. 2.59 [2.50,2.68]), and feeling able to talk about problems with family (Est. 1.49 [1.41,1.56]) were significantly associated with higher FS scores. Additionally, 19 other covariates were found to have some magnitude of significant association. Feeling able to talk about problems with friends had a considerable magnitude of association with higher FS score (Est. 1.63 [1.55,1.71]). While no sex differences were identified in the RE-EM tree, male sex was significantly associated with higher FS score in the LME model (Est. 0.10 [0.04,0.17]), though the magnitude of association was small.

## 4. Discussion

This study used decision trees to examine associations between a range of behavioural and interpersonal risk factors and anxiety, depression, and flourishing outcomes among a large sample of Canadian youth. For all outcomes, the two factors that consistently emerged from the decision trees models as most important were having a happy home life and strong sense of connection to school. The consistency in association seen across three related but distinct measures of mental health provides strong support for the importance of positive home and school environments. Notably, while this study also included a wide array of modifiable behavioural measures that have previously been shown to be related to youth mental health outcomes [30,31,32,33,34] none of these emerged as important in the final tree models. This suggests that interpersonal relationships, particularly those related to home and school environments, are more strongly associated with youth anxiety, depression and flourishing than the individual health behaviours more commonly examined in isolation in the literature. This is important as some characteristics of social support related to school connectedness (SC) and happy home life are potentially modifiable through prevention and intervention efforts by schools and public health professionals. These findings support calls by the MHCC and provincial mental health strategies for prioritization of resources to families and schools for mental health promotion and primary prevention efforts.

The decision tree analysis used in this study is a hypothesis-generating approach in which all available potential risk factors are entered into the models without a priori assumptions. This contrasts with most past research in this field, which has generally taken a hypothesis-testing approach based on theorized associations to a particular risk factor or domain of factors. Despite the difference in approach, the results of the current study align with previous research into the influence of home [24,25,64,65,66,67,68] and school environments [27,29,37,68,69] on youth mental health. However, behavioural factors such as diet, movement behaviors, and substance use which have previously been associated with mental health outcomes [30,31,32,33,34] were not identified as important differentiating factors within the decision tree models in the present study. In fact, the most important factors identified here around social support are typically not included in traditional analyses examining behavioural factors. Given that decision trees also tend to be more parsimonious than regression models in isolating key differentiating factors, the current findings do not necessarily contradict the associations seen in past studies, but rather suggest that the interpersonal relationships from home and school environments are influential factors that require additional consideration in the literature moving forward.

Having a happy home life was identified as the primary distinguishing factor between groups with low and high anxiety and depression scores. Students who indicated not having a happy home life had the highest average GAD-7 scores, with values for females approaching or exceeding the threshold for clinically relevant anxiety symptoms even among those with high SC. Average CESD-10 scores also approached or exceeded the clinical threshold for students of both sexes who indicated not having a happy home life. The influence of the home environment on youth anxiety and depression is well-documented. Past reviews have found consistent associations between parenting style [24,64], interparental conflict [65], and early life stressors [66] on anxiety and depression during adolescence. A review of various sources of social support also found parents and family to be among the most important sources of support to protect against depression in children and adolescents, especially for females [25]. These findings also align with previous decision tree results from Seeley et al. [39] which found family support to be protective among females without previous MDD. In the current study, the home environment was also influential on flourishing: students who indicated having a happy home life had higher average FS scores across all subgroups. While this area of research is newer, these findings are consistent with past studies which have found family resilience and connection to be associated with greater flourishing [67] and adverse family experiences to be associated with lesser flourishing [68] in children and youth. The measure of home environment used in the current study does not provide a definition of the term “happy home life” and is therefore subjective to an individual respondent’s interpretation. Nevertheless, the strong differentiation seen on this measure justifies the need for future validation work to understand how it is interpreted by students. Given that this study also included a measure on feeling able to talk about problems with family, this suggests that the concept of happy home life in relation to mental health is broader than merely the perception of open communication. The perception of happy home life could also be affected by early childhood experiences. While some elements of home life such as parenting style may be considered modifiable through educational interventions, other factors surrounding family dynamic may not be considered modifiable from the perspective of external policymakers and public health professionals. Future work should examine more specifically which aspects of perceived happy home life are contributing to the protective effect seen in this study.

SC was also identified as a key differentiating factor across all outcomes, highlighting the importance of a positive school environment to youth mental health. Past research has similarly found SC and belonging to be protective against depression [27,29]. In the current study, SC was protective among students without a happy home life; average GAD-7 scores were at or below the clinical threshold for those who had high SC, compared to exceeding the threshold for those with low SC. Average CESD-10 scores were also over 4 points lower for those with high SC among both sexes without a happy home life. This is consistent with past research which found that SC moderated the relationship between family obligations and emotional distress among middle and high school students [23]. SC was also identified as the primary distinguishing factor between groups with low and high FS scores. Smaller studies have found consistent associations between sense of school community or belonging with measures of wellbeing [69,70]. This finding has important implications for school-based interventions since it suggests that schools can play a meaningful role in increasing mental wellbeing among students—even among those who may not have a happy home life—by cultivating a climate of connection and belonging. Further research into evidence-based policy and program interventions for increasing school connection is warranted.

Consistent with literature regarding adolescent mental illness prevalence [1], differences by sex were identified for anxiety and depression outcomes in the decision trees, with female subgroups having consistently higher average GAD-7 and CESD-10 scores than corresponding male subgroups. These differences are commonly posited to be related to sociocultural gender norms [1], with females being more likely than males to exhibit internalizing symptoms [71,72]. Notably, no differences by sex emerged in the decision tree for flourishing. This is an important finding in the context of school-based intervention as it suggests that males and females could benefit equally from initiatives to increase school connection. Other demographic factors such as ethnicity and age were found to be associated with mental health outcomes in the LME models but not the decision tree models.

While the decision tree results provide insight into distinguishing factors and high-risk subgroups, the LME results describe the average effect of each factor on the total sample after controlling for all other factors. The LME models in this study had higher fit indices, as measured by R^2^_adj_, than the corresponding tree models but were also much more complex. Notably, R^2^_adj_ was at or below 50% for all models, which is unsurprising given a likely genetic component to youth mental health outcomes that cannot be explained by environmental factors. For most variables identified as statistically significant in the LME models but not included in the corresponding decision trees, the LME magnitude of association was small. One exception to this was having been bullied in the past 30 days, which had a large magnitude of association with anxiety and depression outcomes. Post hoc *t*-tests found a moderately strong negative association between bullying and SC (*p* < 0.0001, Cohen’s d 0.588), suggesting that the impact of bullying on groups with higher GAD-7 and CESD-10 scores may already be accounted for through differentiation on SC in the tree models. This hypothesis is supported by previous studies which have found SC to be a mediating factor in the relationship between bullying and mental health indicators [73,74]. Aside from this factor, the decision trees captured the key distinguishing factors in more parsimonious and easily interpretable and flexible models than LME, allowing for effective knowledge translation. Decision trees also identified underlying non-linear associations for SC, as can been seen by this factor being split recursively across different cut points. This highlights the ability of decision trees to capture complex relationships that are often missed when using standard regression analysis.

### Strengths and Limitations

This is one of the first studies to use decision tree methods to examine youth depression, anxiety, and flourishing outcomes and associated behavioural and interpersonal risk factors. Unlike past research which commonly used regression approaches, the use of decision trees allows for the identification of key differentiating factors and high-risk subgroups. This study also used hierarchical RE-EM trees which properly account for the clustered nature of the data and are novel to public health research. However, decision tree techniques have limitations, including lower prediction accuracy than other methods, and a tendency to overfit the sample data which is only partially mitigated by pruning. Additionally, while this study benefits from a large sample size, the sampling method used is not representative and therefore results may not be reflective of all Canadian youth. Additionally, this study uses self-report data and thus mental health indicators are not based on clinical assessment. Further, this study is cross-sectional and thus temporality between risk factors and mental health outcomes cannot be inferred. Notably, perceptions of happy home life and school connection may be consequences of or bi-directionally associated with mental health status. Further longitudinal research into the directionality of associations is warranted. Lastly, some measures used in this study contained meaningful amounts of missing data, which could introduce bias if missingness does not occur completely at random. LME models use maximum likelihood estimation and are unbiased when outcome missingness can be explained by the observed covariates; however, this assumption is untestable. RE-EM trees handle missing covariate data using surrogate splits but cannot correct for missing outcome data. Mental health scales were person-mean imputed for students missing 1 or 2 items to partially recover missing responses. Multiple imputation has been suggested as the preferred approach to handle missing data in Likert-type scales such as the CESD [75]; however, there is limited research into how to apply multiply imputed datasets to the generation of a single interpretable decision tree.

## 5. Conclusions

This study found that, across a range of interpersonal and behavioural factors, having a happy home life and SC were key differentiators of youth anxiety, depression, and flourishing levels. This highlights the importance of the influence of home and school environments on youth mental health and supports calls for national policy focus and investment in family and school resources. While having a happy home life is most important in protecting against youth anxiety and depression, a sense of connection to school may mitigate the negative influence of a poor home environment. Schools can also play a meaningful role in contributing to positive mental health among students by cultivating a sense of belonging.

## Figures and Tables

**Table 1:** Sample characteristics for students participating in Year 7 (2018–2019) of the COMPASS Study (*N* = 74,501).

Continuous Variables	Mean (s.d.)
CESD-10 [*N* = 70,610]	8.82 (6.05)
GAD-7 [*N* = 71,736]	6.2 (5.56)
FS [*N* = 72,415]	32.16 (5.73)
Age [*N* = 73,960]	15.15 (1.49)
School Area Median Income (‘000s) [*N* = 74,501]	67.59 (17.45)
School Size (‘00s) [*N* = 74,501]	8.41 (3.52)
Servings of Fruits and Vegetables [*N* = 71,679]	2.98 (2.01)
Average Daily Physical Activity (h) [*N* = 66,007]	1.6 (1.05)
Screen Time (h) [*N* = 67,181]	5.87 (3.04)
Sleep Time (h) [*N* = 69,630]	7.52 (1.28)
School Connectedness Score [*N* = 71,413]	18.5 (3.36)
**Binary Variables**	**% (*n*)**
Eat Breakfast Daily [*N* = 74,501]	0.49 (36,197)
Tobacco Use [*N* = 73,852]	0.07 (5532)
E-cigarette Use [*N* = 73,466]	0.28 (20,852)
Binge Drinking [*N* = 74,254]	0.17 (12,884)
Cannabis Use [*N* = 73,299]	0.13 (9662)
Was Bullied [*N* = 70,753]	0.88 (61,940)
Bullied Others [*N* = 71,063]	0.06 (4339)
Expect to Attend Post Secondary Education [*N* = 70,753]	0.06 (4339)
Happy Home Life [*N* = 72,830]	0.79 (57,444)
Talk About Problems with Family [*N* = 72,234]	0.59 (42,833)
Talk About Problems with Friends [*N* = 72,622]	0.75 (54,246)
**Categorical Variables**	**% (*n*)**
Sex [*N* = 73,672]	
*Female*	0.5 (36,546)
*Male*	0.5 (37,126)
Ethnicity [*N* = 73,839]	
*White*	0.69 (51,017)
*Black*	0.04 (2951)
*Asian*	0.1 (7465)
*Hispanic*	0.03 (1886)
*Other/Mixed*	0.14 (10,520)
Spending Money [*N* = 73,422]	
*$* *0*	0.16 (11,684)
*$* *1–* *$* *20*	0.24 (17,744)
*$* *21–* *$* *40*	0.11 (8071)
*$* *41–* *$* *100*	0.12 (8722)
*More than* *$* *100*	0.19 (14,216)
*Don’t Know*	0.18 (12,985)
Province [*N* = 74,501]	
*AB*	0.04 (3301)
*BC*	0.14 (10,402)
*ON*	0.41 (30,675)
*QC*	0.4 (30,123)
Urbanicity [*N* = 74,501]	
*Large Urban*	0.54 (40,421)
*Medium Urban*	0.1 (7573)
*Small Urban/Rural*	0.36 (26,507)
Weight Perception [*N* = 73,071]	
*Underweight*	0.17 (12,140)
*About the right weight*	0.6 (43,893)
*Overweight/Obese*	0.23 (17,038)
BMI Classification [*N* = 74,501]	
*Underweight*	0.02 (1397)
*Normal Weight*	0.53 (39,388)
*Overweight*	0.12 (8682)
*Obese*	0.05 (4027)
*Not Stated*	0.28 (21,007)
Classes Skipped in Past 4 Weeks [*N* = 71,571]	
*0 classes*	0.65 (46,785)
*1 or 2 classes*	0.2 (14,555)
*3 to 5 classes*	0.08 (5988)
*6 or more classes*	0.06 (4243)

*N* = number of non-missing responses to questionnaire measure; s.d. = standard deviation.

**Table 2:** Linear Mixed Effects Models for GAD-7, CESD-10, and FS Outcomes among students in Year 7 (2018–2019) of the COMPASS Study.

[Estimate (95% Confidence Interval)]		GAD-7 (*N* = 52,875)	CESD-10 (*N* = 52,591)	FS (*N* = 52,997)
Male Sex		−2.57 (−2.65,−2.49) ***	−2.11 (−2.19,−2.03) ***	0.1 (0.04,0.17) **
Age		0.09 (0.06,0.12) ***	0.06 (0.02,0.09) ***	−0.06 (−0.09,−0.03) ***
Ethnicity (ref = White)	Black	−1.54 (−1.77,−1.31) ***	−1 (−1.24,−0.76) ***	1.1 (0.91,1.3) ***
	Asian	−0.69 (−0.84,−0.53) ***	0.15 (−0.01,0.3)	−0.43 (−0.56,−0.3) ***
	Hispanic	−0.4 (−0.65,−0.15) **	−0.08 (−0.34,0.19)	0.6 (0.38,0.81) ***
	Other/Mixed	−0.02 (−0.14,0.09)	0.17 (0.04,0.29) **	0.19 (0.09,0.29) ***
Spending Money (ref = $0)	$1–$20	−0.05 (−0.17,0.07)	0 (−0.13,0.13)	0.35 (0.24,0.45) ***
	$21–$40	−0.29 (−0.44,−0.14) ***	−0.2 (−0.36,−0.05) *	0.58 (0.45,0.71) ***
	$41–$100	−0.16 (−0.31,−0.01) *	−0.19 (−0.34,−0.03) *	0.66 (0.53,0.79) ***
	More than $100	−0.08 (−0.22,0.06)	−0.35 (−0.49,−0.2) ***	0.87 (0.75,0.99) ***
	Don’t Know	−0.22 (−0.36,−0.09) ***	−0.25 (−0.39,−0.11) ***	0.37 (0.26,0.49) ***
Province (ref = AB)	BC	−0.11 (−0.48,0.26)	NI	−0.56 (−0.91,−0.21) **
	ON	−0.07 (−0.38,0.24)	NI	−0.29 (−0.59,0.01)
	QC	−0.61 (−0.93,−0.29) ***	NI	0.21 (−0.1,0.52)
Urbanicity (ref = Large Urban)	Medium Urban	−0.02 (−0.26,0.22)	−0.23 (−0.47,0.02)	NI
	Small Urban/Rural	−0.29 (−0.44,−0.14) ***	−0.26 (−0.41,−0.1) **	NI
School Size (‘00s)		NI	NI	0.03 (0.01,0.05) **
Weight Perception (ref = Underweight)	About the right weight	−0.57 (−0.68,−0.46) ***	−0.6 (−0.72,−0.49) ***	0.37 (0.27,0.46) ***
	Overweight/Obese	0.23 (0.09,0.36) **	0.34 (0.2,0.48) ***	−0.6 (−0.71,−0.48) ***
BMI Classification (ref = Underweight)	Normal Weight	−0.02 (−0.31,0.26)	0.13 (−0.17,0.42)	−0.22 (−0.46,0.02)
	Overweight	−0.29 (−0.6,0.01)	−0.05 (−0.37,0.27)	0.08 (−0.18,0.35)
	Obese	−0.17 (−0.5,0.17)	0.09 (−0.26,0.43)	0.09 (−0.19,0.38)
	Not Stated	−0.13 (−0.42,0.16)	0.03 (−0.28,0.33)	−0.39 (−0.64,−0.15) **
Eat Breakfast Daily		−0.57 (−0.65,−0.48) ***	−0.77 (−0.86,−0.69) ***	0.31 (0.24,0.38) ***
Servings of Fruits and Vegetables		0.06 (0.04,0.08) ***	0.04 (0.01,0.06) ***	0.14 (0.12,0.15) ***
Average Daily Physical Activity (h)		0.05 (0.01,0.08) *	NI	0.43 (0.4,0.47) ***
Screen Time (h)		0.1 (0.09,0.12) ***	0.11 (0.1,0.13) ***	−0.07 (−0.08,−0.06) ***
Sleep Time (h)		−0.43 (−0.47,−0.4) ***	−0.6 (−0.64,−0.56) ***	0.31 (0.28,0.34) ***
Tobacco Use		0.2 (0.02,0.39) *	0.46 (0.27,0.65) ***	−0.12 (−0.27,0.03)
E-cigarette Use		0.2 (0.1,0.3) ***	0.39 (0.29,0.5) ***	NI
Binge Drinking		NI	NI	0.25 (0.15,0.35) ***
Cannabis Use		0.15 (0,0.29) *	0.16 (0.01,0.31) *	NI
Was Bullied		1.92 (1.79,2.05) ***	2.05 (1.93,2.18) ***	−0.47 (−0.58,−0.36) ***
Bullied Others		0.19 (0.01,0.37) *	NI	−0.56 (−0.71,−0.4) ***
Expect to Attend Post-Secondary Education		0.41 (0.32,0.51) ***	−0.18 (−0.28,−0.08) ***	0.63 (0.55,0.71) ***
Classes Skipped in Past 4 Weeks (ref = 0 classes)	1 or 2 classes	0.26 (0.16,0.36) ***	0.36 (0.25,0.46) ***	−0.09 (−0.18,−0.01) *
	3 to 5 classes	0.48 (0.34,0.63) ***	0.6 (0.44,0.75) ***	−0.18 (−0.3,−0.05) **
	6 or more classes	0.72 (0.54,0.91) ***	0.95 (0.76,1.14) ***	−0.24 (−0.4,−0.09) **
School Connectedness Score		−0.32 (−0.33,−0.3) ***	−0.45 (−0.46,−0.43) ***	0.68 (0.67,0.69) ***
Happy Home Life		−1.96 (−2.07,−1.85) ***	−2.75 (−2.86,−2.64) ***	2.59 (2.5,2.68) ***
Talk about Problems with Family		−0.84 (−0.92,−0.75) ***	−1.31 (−1.4,−1.22) ***	1.49 (1.41,1.56) ***
Talk about Problems with Friends		−0.6 (−0.69,−0.51) ***	−0.85 (−0.95,−0.76) ***	1.63 (1.55,1.71) ***

* *p* < 0.05, ** *p* < 0.01, *** *p* < 0.001, NI = variable not included in final model after backward selection.

## Data Availability

Data are not publicly available while the study is ongoing. Data are available upon request to the COMPASS Study Principal Investigator: https://uwaterloo.ca/compass-system/information-researchers accessed on 13 July 2022.
